# The role of European chemical manufacturing companies in promoting effective communication of conditions of safe use by workers

**DOI:** 10.1093/annweh/wxae042

**Published:** 2024-05-18

**Authors:** Jan Urbanus, Evelyn Tjoe Nij, Cornelia Tietz

**Affiliations:** Product Stewardship, Belgian Shell NV, Brussels, Belgium; Product Regulatory Services EMEAI, DOW Benelux BV, Terneuzen, The Netherlands; European Solvents Industry Group, Brussels, Belgium

**Keywords:** chemical safety, chemicals regulation, REACH, Safe Use Communication, Safety Data Sheets

## Abstract

In 2006, the revised chemicals management legislation mandated that manufacturers of hazardous chemical substances conduct risk assessments for the entire substance life cycle. Additionally, they must communicate use-specific safe handling advice (exposure scenarios) to their customer, as annex to the Safety Data Sheet (SDS). Despite significant efforts to develop workable solutions for chemical mixtures, this goal has not yet been fully achieved. Therefore, a Cefic research project (LRI B23) was commissioned on how to ensure meaningful health risk communication for workers across supply chains. The research project determined that risk-based safe use advice generated by manufacturers, often does not reach the intended end-user and was seen as not tailored to specific user needs. Recipients of the advice are also not prepared to act based on information developed by suppliers. From an industry perspective, the complexity of supply chains and substance life cycles are considered major barriers for effective safe use communication. Exposure scenarios for substance use in industrial work environments are often perceived as adding little value compared to existing safe use arrangements required by other health, safety, and environmental legislation applicable to employers and duty-holders. To attain meaningful use-specific safe handling advice for workers, including those at non-industrial premises who may benefit most from such advice, knowledge transfer and close collaboration between manufacturers and formulators remain key elements, supported by enhanced regulatory appreciation.

## Introduction

The European Regulation (EC) No 1907/2006 concerning the Registration, Evaluation, Authorisation and Restriction of Chemicals (REACH) introduced the legal requirement on chemical manufacturers of hazardous chemical substances to communicate, for the entire substance life cycle, use-specific safe handling advice in the form of exposure scenarios (ES) annexed to the Safety Data Sheet (SDS).

Despite years of substantial efforts, many stakeholders still consider ES as adding little practical value to improve the safe use of chemicals in the workplace.

The mandated information exchange processes have mostly been established via sector organizations and REACH consortia of registrants to create some level of uniformity across supply chains. Still, some essential elements, like the link with existing occupational safety and health (OSH) requirements, communication aspects, and management of change were overlooked.

A reaction to the findings of the LRI B23 project (Otten et al. 2022) and the experiences and barriers encountered by the industry are described in this paper. The aim is to open the discussion on a different but more effective safe use communication.

### Original intent of the legislator

Via REACH, Europe intended to reinforce the risk-based approach for safe handling of hazardous chemicals, both from an environmental and human health perspective. The advent of this regulation and, in particular, the requirement to communicate the appropriate risk management measures for the safe handling for each substance application, created a promising novel angle. In Europe, workers would no longer be at risk of developing occupational diseases from excessive exposure to chemicals due to a lack of appropriate safe handling advice.

Article 37 of REACH stipulates that downstream users are required to apply the appropriate measures to control the risk, meaning that compliance with the ES received is required. To facilitate the creation of meaningful ES, the use information of substances (as such or as part of a mixture) must be passed up the supply chain by downstream users so that the registrant can develop suitable ES. The ES received from the supplier becomes complementary to the OSH duties on each employer to assess and manage health risks for the employed workers. How REACH and OSH risk assessments cohabitate, from an industry perspective, is described in a Cefic paper (Cefic 2021).

### Experiences and barriers

In the early days of REACH, in the absence of consolidated guidance, registrants mainly used in-house developed solutions in top-down approaches for the ES, due to the extreme time pressure set by the legislation for the first large wave of substances to be registered. In addition, the approach was, on purpose, generic to cover also downstream uses that had not yet been identified. Representatives of manufacturing companies decided to communicate the same ES developed for the REACH registration dossiers (Money et al. 2011).

A simplistic view of a supply chain has as starting point a chemicals manufacturing or importing company. A substance is sold to another company, which can be a formulator, who mixes several chemical substances to make a performance product, for example, a coating or a cleaning agent. The various chemicals making up the mixture have different technical functions (e.g. solvent, pigment, detergent), and the proportions of the substances have been chosen to obtain the required performance upon application by the end user of the product (e.g. a painter or a cleaner). Just like in product formulation, also the information on the safe use of the substances making up the product must be assembled and processed into safe handling instructions for the final product.

In reality, supply chains and substance life cycles are much more complex, have business-confidentiality aspects, and are subject to frequent change. The first level of downstream user can be a distributor, which co-mingles supplies of the same substance from different producers (registrants) and relabels the substance as its own. Formulators further in the supply chain take the initial mixture together with other substances or mixtures to create a new mixture. Equally, many downstream users procure their chemical products from multiple suppliers. Although the intent has always been to have ES in one joint registration dossier and that these should be communicated and updated in a coordinated way, in practice, there is a large variability in quality, format and content.

It is this complexity, which was already identified shortly after the introduction of the regulation, that has proven hard to navigate for a safe use information flow up and down the supply chain. Furthermore, there are typically multiple downstream users at each level of the supply chain, each with their own, somewhat different conditions of use. Also, the hierarchy of control, a well-known principle in health and environmental risk management, and required by national OSH regulations (Chemicals Agents Directive 98/24/EC, article 6), is not always adhered to when stipulating risk management measures. In addition, insights have emerged that the precision of the predictions generated with a screening-level exposure assessment tool (a so-called Tier-1 tool) is rather low for specific workplaces (Savic et al. 2023). The generic system of use descriptors and accompanying tier-1 exposure assessment models do not allow for the required higher level of granularity, as also indicated in a progress report on REACH implementation (ECHA 2021).

With the Lead Component Identification (LCID) method, the manufacturing industry developed guidance on how substance information can be reconciled for mixtures (Cefic-VCI 2018). For the implementation of the LCID methodology, formulators rely on many data points. With updates of substance chemical safety reports (CSRs), the data points for individual ingredients frequently change, making it very challenging to have up-to-date mixture safe use information at the end of the supply chain. Typically, mixtures are added to mixtures, creating another level of complexity, especially when those mixtures are purchased from outside the EU. A registrant can also not ascertain for himself that the ES constructed for the chemical's use at secondary or tertiary actors’ premises are indeed appropriate. Mixture safe use information is only required at the end of the supply chain if classified substances are present in end-user products at concentrations at or above the mixture classification cut-off for the particular hazard (typically 0.1% or 1%, as defined in the regulation on Classification, Labelling and Packaging of substances and mixtures (CLP - (EC) No 1272/2008). If the registrant is unaware that the end product is not classified, this may result in unnecessary communication down the supply chain. The theoretical concept of combining risk assessments from individual ingredients to mixture safe use advice presents numerous practical challenges, especially in the absence of a single method to process the mixture component information (Taxel et al. 2014). They also found risk management measures for mixtures to be over-precautionary. In practice, ES are often annexed only to the substance SDS and not further communicated down the supply chain when multiple substances are combined into a mixture.

A series of improvements were proposed via the Exchange Network on Exposure Scenarios (ENES), a collaborative platform of regulators and industries created in 2011 and active till 2019, to identify chemical end-user information needs with a primary role of formulators of products made from multiple chemicals. Not all improvements were successfully implemented.

### Findings

In view of the observed “gap-to-potential”, a research project was defined in 2020 and funded under Cefic’s Long-range Research Initiative (LRI) (Cefic-LRI 2019). An independent, multidisciplinary research consortium conducted surveys, interviewed many actors in the supply chain, and published their findings in a report (Otten et al. 2022) and in the scientific literature (Fransman et al. 2023).

One of the principal conclusions from this project is that the safe use advice generated at REACH registrant level often does not reach the intended end-user, as ES are generally not available on mixture SDS. For substances, ESs are often considered too long, too generic, and not tailored to specific user conditions, and equally too complex for technologically less advanced actors. ES are based on a generic use descriptor system and mostly exposure assessment models and are intended to conservatively encompass as many workplaces as possible.

It was noted that end-users primarily use chemical products that are mixtures, for which there is no legal requirement to create ES. These end-users have so far been largely absent from the deliberations; hence, sector expertise is lacking. Also, end users tend not to differentiate between different regulations such as REACH, OSH, and CLP. It was also noted that whilst REACH is an EU-wide legislation, the surrounding area (legislation, organizations, and culture) is more national, which influences how REACH is perceived and implemented in practice. The Cefic LRI project explored among others, the aspect of persuasion, i.e. how the recipient views the messenger (i.e. the supplier's extended SDS) and is prepared to accept and implement the advice. Although legally required, the provision of ES via SDS is seen as less than ideal. An opinionated Dutch publication (Tjoe Nij et al. 2022) describes an exercise that concluded that professionals in the field of occupational hygiene can consistently identify the “Identified use,” but that the interpretation of the content of the ES is variable among peers. Recommendations from the LRI B23 project directed to the chemical manufacturing companies are summarized in Table 1.

**Table 1.** Research recommendations from LRI B23 for chemical manufacturing companies.

Recommendation	Reflection from authors
Ensure REACH documents are accompanied by understandable information adapted to intended audience; Use techniques from social sciences to improve uptake of safe handling advice	Registrants alone cannot solve this—need Downstream User steer (formulator with end-user input; national/regional industry) including formats, modalities (e.g. training course), existing avenues
Digitisation of documentation for ease of transfer	Requires an agreed and complete standardization of data elements and formats
Facilitate upstream information flow by actively asking feedback; standardize feedback reporting; Encourage end-users to communicate (up) information on safe handling	Requires novel approaches; role for sector organizations?
Respect the hierarchy of control when specifying risk management measures	Minimum content quality requirements for REACH exposure scenario creation—involve appropriate in-house expertise in the development of exposure scenarios

### Alternative approaches

Some alternative approaches have been elaborated in the course of ENES. Sector associations have developed so-called use maps and Safe Use of Mixtures Information cards (SUMIs). The latter has been implemented at the product level, independent of substance ES, by some sectors (e.g. the Soaps and Detergents sector) (AISE 2018). The use maps proposed by downstream user sector organizations are chemically agnostic, i.e. they do not identify specific chemicals for which the upstream registrants can readily identify the need to incorporate the information in their dossiers.

## Discussion

When reflecting on all the work done both by industry and regulators to provide risk-based safe use advise for chemical products, it is clear that the ambition was high, and the complexity was initially not fully understood. The frequently changing regulatory requirements, use and tonnage information, and hazard conclusions make it very challenging to communicate up-to-date ES in the supply chain for substances and even more so for mixtures. The communication of ES as an annex to a substance SDS does not appear to be an effective avenue to meet the intent of the REACH regulation with respect to worker health protection and may be at odds with OSH requirements. From purely a worker health perspective, it seems redundant to provide risk-based safe use advise for substances via an ES, as formulators and users of substances and mixtures are already required to do their own risk assessments by experts in the field under national OSH regulations. Information provided in the main body of the SDS, suffices as a basis for good workplace risk assessment. For professional workers, who mainly use mixtures, no viable fit-for-all sectors solution has emerged so far.

Today via its trade association Cefic and its Network of Experts for Safe Use Communication, European chemical manufacturing companies together with the main downstream user associations, attempt to address several of the recommendations from the LRI B23 project.

However, it appears that fundamental re-thinking is required to achieve effective communication of safe use advice for workers.

The authors see a credible and sustainable solution. The, by now traditional, approach to occupational risk management is based on the hierarchy of control. Occupational hygienists advise the elimination or substitution of the hazardous chemical in a product or process, closed processes, local exhaust ventilation or general ventilation, administrative controls, and finally personal protective equipment. Although this concept has also been advocated for REACH required substance risk assessment, this dogmatism is often in conflict with the conditions required to reach the intended performance of a product or process and thus becomes moot. It is clear that exposure control cannot be left to the discretion of the chemical product users and their occupational hygienist but must become integral to product design at the level of the product formulator. Such a systems approach should be promoted by assigning formal responsibility to product formulators to integrate exposure control into the technical performance characteristics of products containing classified chemical substances. The role of the practicing occupational hygienist then becomes the verification of the working conditions and their effectiveness as intended by the product designers, rather than operating in a vacuum as so often still happens today. Vice versa, product designers should actively seek the dialogue with exposure control specialists. Exposure chamber studies and advanced exposure modelling can play an important role in product development to ensure that postulated risk management measures are realistic. This is also the phase to consider the elimination or substitution question.

Promoting and disseminating such good practice can be the role of formulator sector organizations and branch organizations, at European, national, or regional levels, whichever is most persuasive to the end users.

The implication of all of this for registrants (manufacturers or Importers) under REACH and their initial downstream users such as distributors, is that, for uses that are determined to be safe in the Chemical Safety Report, listing the “Identified Uses” in section 1 of the SDS, and including references to such sector documents should be sufficient. This way, confusion from untargeted exposure control advice (ES) in supply chains and by product end users can be avoided.

The REACH toolbox can also be used effectively in the more holistic Safe and Sustainable by Design methodology, a key element of the transition pathway of for the European Chemical Industry. Again, seeking and encouraging active dialogue is key to the sustainable use of chemicals without undesired health consequences.

## Data Availability

No data were used in this study.
